# Idiopathic granulomatous mastitis after mRNA vaccination against COVID-19: a possible association?

**DOI:** 10.1093/bjrcr/uaae048

**Published:** 2025-02-20

**Authors:** Elisenda Vall, Vicente Araya, Lidia Tortajada, Vanessa Escobedo, Rosa Nogueiras, Javier del Riego

**Affiliations:** Women’s Imaging, Department of Radiology, UDIAT Centre Diagnòstic, Parc Taulí Hospital Universitari, Institut d’Investigació i Innovació Parc Tauli I3PT, Univertitat Autònoma de Barcelona, Sabadell, Barcelona, 08201, Spain; Women’s Imaging, Department of Radiology, UDIAT Centre Diagnòstic, Parc Taulí Hospital Universitari, Institut d’Investigació i Innovació Parc Tauli I3PT, Univertitat Autònoma de Barcelona, Sabadell, Barcelona, 08201, Spain; Women’s Imaging, Department of Radiology, UDIAT Centre Diagnòstic, Parc Taulí Hospital Universitari, Institut d’Investigació i Innovació Parc Tauli I3PT, Univertitat Autònoma de Barcelona, Sabadell, Barcelona, 08201, Spain; Department of Pathology, Parc Taulí Hospital Universitari, Institut d’Investigació i Innovació Parc Tauli I3PT, Univertitat Autònoma de Barcelona, Sabadell, Barcelona, 08201, Spain; Department of Gynaecology, Parc Taulí Hospital Universitari, Institut d’Investigació i Innovació Parc Tauli I3PT, Univertitat Autònoma de Barcelona, Sabadell, Barcelona, 08201, Spain; Women’s Imaging, Department of Radiology, UDIAT Centre Diagnòstic, Parc Taulí Hospital Universitari, Institut d’Investigació i Innovació Parc Tauli I3PT, Univertitat Autònoma de Barcelona, Sabadell, Barcelona, 08201, Spain

**Keywords:** breast imaging, mastitis, breast MRI, COVID-19

## Abstract

Idiopathic granulomatous mastitis (IGM) is an uncommon benign disease thought to have an autoimmune origin. After massive vaccination against COVID-19, mRNA vaccines have been associated with various possible adverse effects. Among those involving the breast, the most common are ipsilateral axillary lymphadenopathies and transient breast oedema. We present the case of a young woman who developed IGM after mRNA vaccination against COVID-19. We describe the clinical and imaging findings and management of this case, discussing the evidence for a possible link between vaccination and the development of this uncommon inflammatory process and underlining the importance of including this entity in the differential diagnosis in this scenario.

## Background

Also known as non-puerperal mastitis, idiopathic granulomatous mastitis (IGM) is a benign inflammatory disease with a chronic (persistent or recurrent) course that occurs mainly in women of childbearing age.^1^ IGM is uncommon (<1% of breast biopsies) and has no known ethnic predominance. Although the aetiology is unknown, the prevailing theory is that IGM is an autoimmune disease. The inflammatory response can be triggered by multiple factors, such as drugs, diabetes, trauma, and smoking, but the only factors that have been strongly associated with IGM are pregnancy, lactation, and hyperprolactinaemia.^2^

The clinical presentation and imaging findings for IGM can mimic those of breast cancer. IGM most commonly presents as a palpable mass in association with mild erythema and variable local pain.^1^ Because the clinical and imaging findings are non-specific, the diagnosis requires histological confirmation.

New mRNA vaccines for COVID-19 trigger an immune response that can result in both local and systemic adverse effects; patients can develop episodes of relapse of previous autoimmune disease or develop a new autoimmune or autoinflammatory condition.^3^ Nevertheless, the causal relationship between COVID-19 vaccines and these autoimmune diseases remains to be demonstrated.^4^

To our knowledge, no cases of IGM after COVID-19 vaccination have been reported. We present the case of a patient who developed IGM following vaccination against COVID-19. We describe the clinical and imaging features of this disease and provide guidelines for managing it in this scenario.

## Case presentation

**Figure 1.**
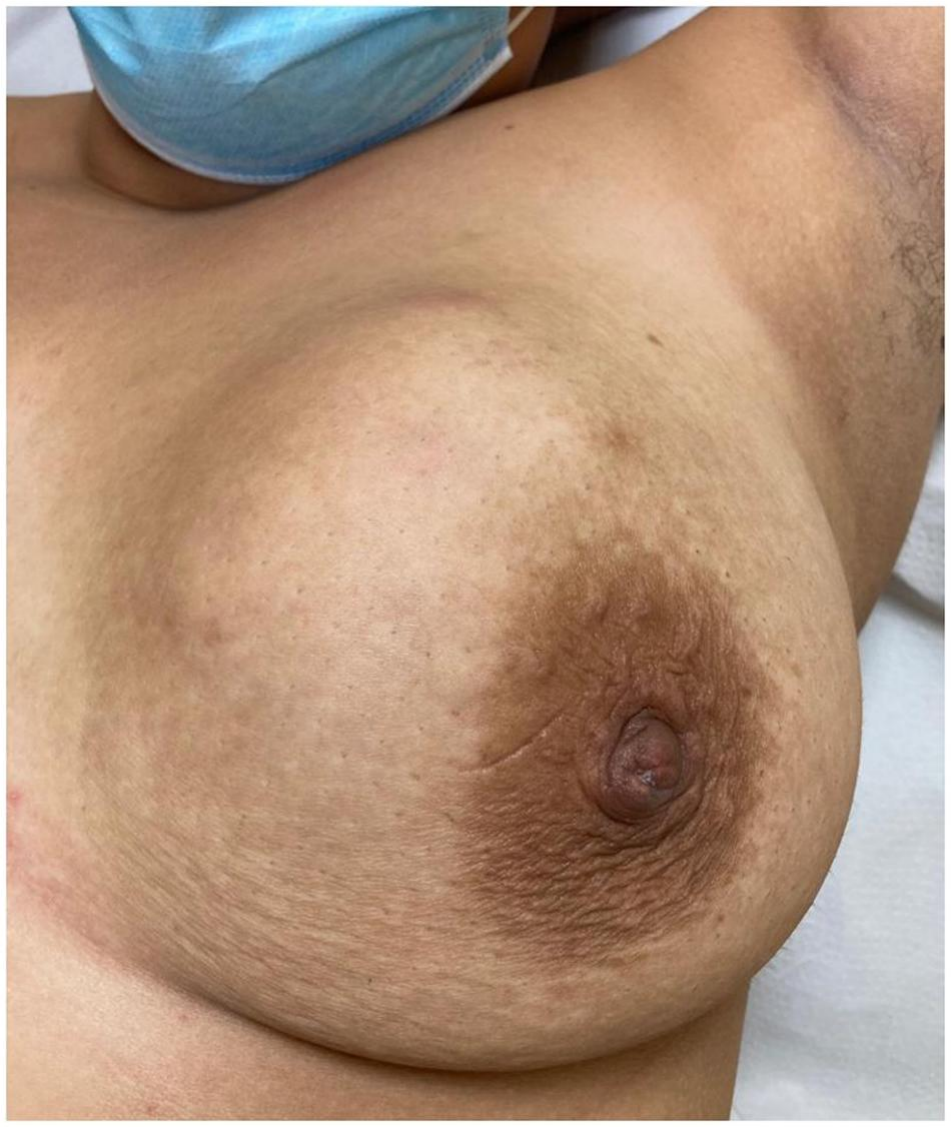
Photograph showing a voluminous indurated mass in the upper-inner quadrant of the left breast, with associated areas of mild erythema.

The patient was a 24-year-old smoker who had given birth once at the age of 21. After a normal vaginal delivery, she breastfed the baby for 18 months without complications. She presented with an indurated palpable mass that had appeared suddenly in the upper-inner quadrant of her left breast with associated areas of mild erythema 48 h after being vaccinated against COVID-19 (BNT162 mRNA COVID-19 vaccine; Pfizer-Biontech, Mainz, Germany) and had progressed rapidly within 36 h (Figure 1).

**Figure 2.**
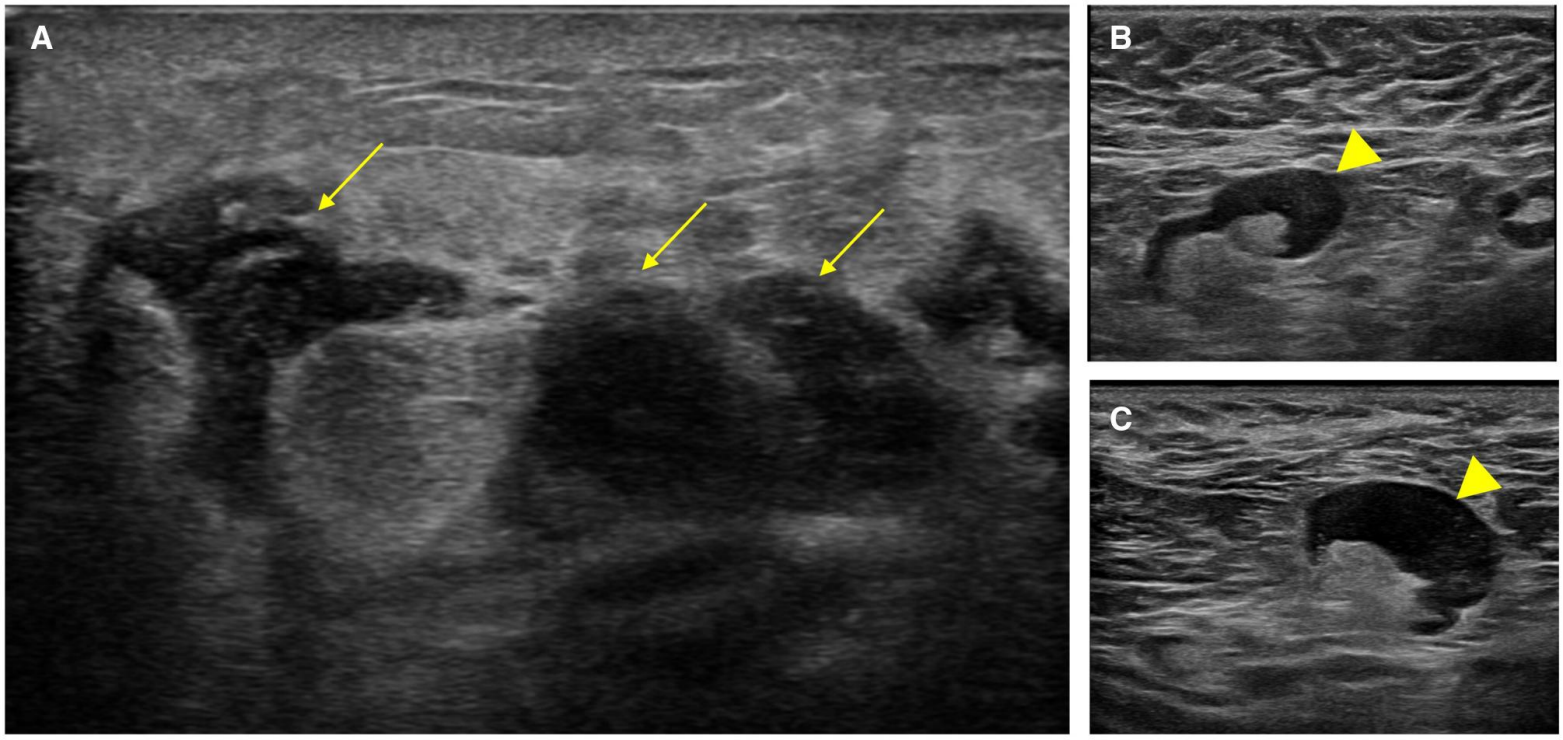
Ultrasound of the breast and axilla. (A) Underlying the palpable mass in the left breast, there is a large ill-defined heterogeneous zone with multiple irregular hypoechogenic areas and posterior acoustic shadow (arrows). (B, C) Multiple adenopathies with focal cortical thickening are seen in level I of the ipsilateral axillary lymph node chain (arrowheads).

**Figure 3.**
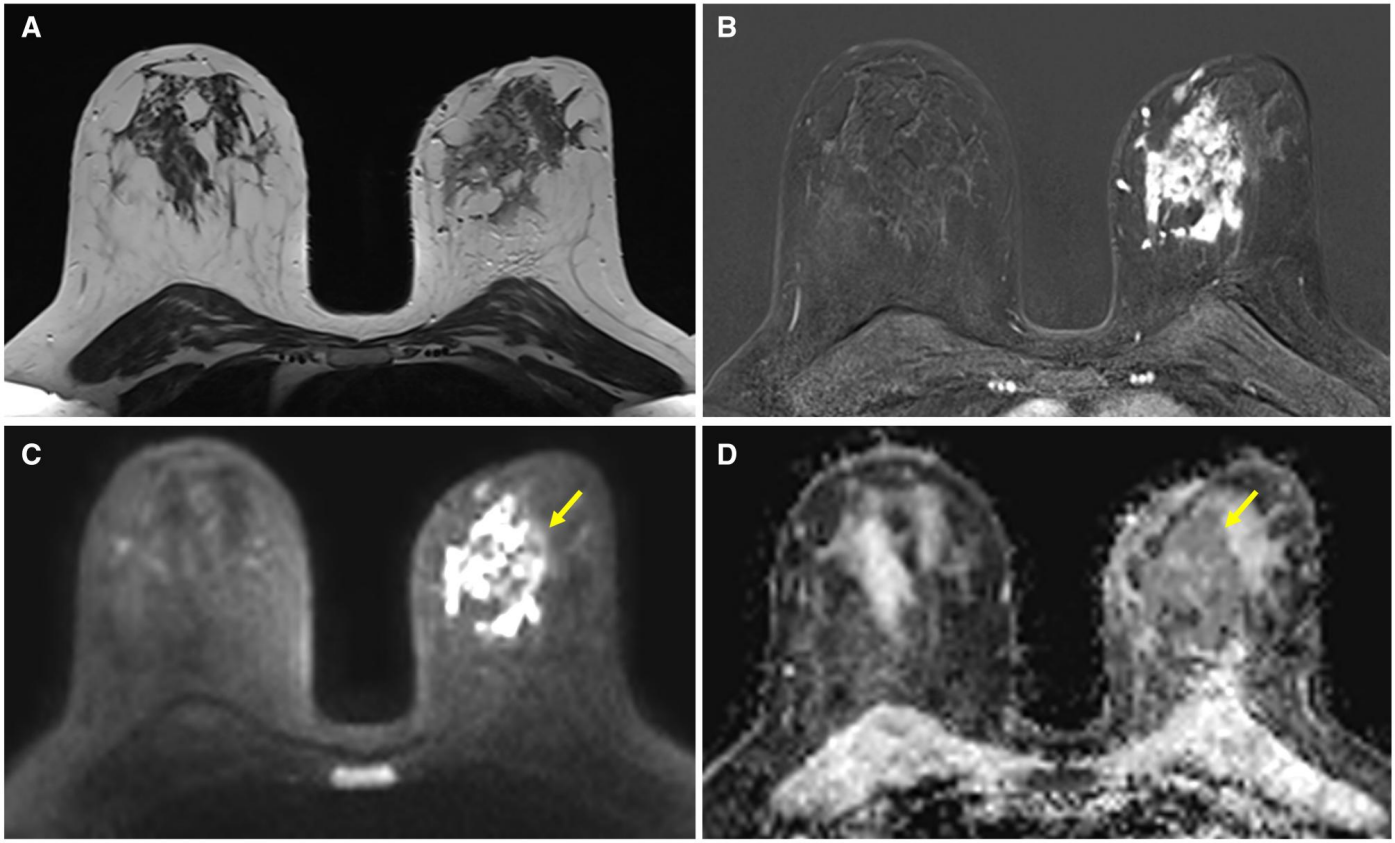
Breast MRI. (A) Axial T2-weighted, (B) axial contrast-enhanced T1-weighted spoiled gradient-echo (subtracted image obtained 120 s after contrast injection), (C) axial diffusion-weighted imaging (b-800 value), and (D) axial apparent diffusion coefficient (ADC) maps show an extensive area of heterogeneous non-mass-like enhancement in the internal quadrants of the left breast in multiple regions and restricted diffusion (arrows). ADC = 0.83 × 10^−3^ mm^2^/s. Note how MRI enables a better assessment of the overall extent of the lesion.

Bilateral breast ultrasound showed an extensive ill-defined heterogeneous area with multiple irregular hypoechogenic lesions with posterior acoustic shadowing underlying the mass, as well as 4 enlarged lymph nodes with focal cortical thickening in her left axilla (Figure 2). No established collections were observed. Bilateral breast MRI showed an extensive area of heterogeneous non-mass-like enhancement distributed in multiple areas in nearly the entire volume of the inner quadrants of the left breast; on diffusion-weighted imaging, these areas showed restricted diffusion (apparent diffusion coefficient: 0.83 × 10^−3^ mm^2^/s) (Figure 3).

**Figure 4.**
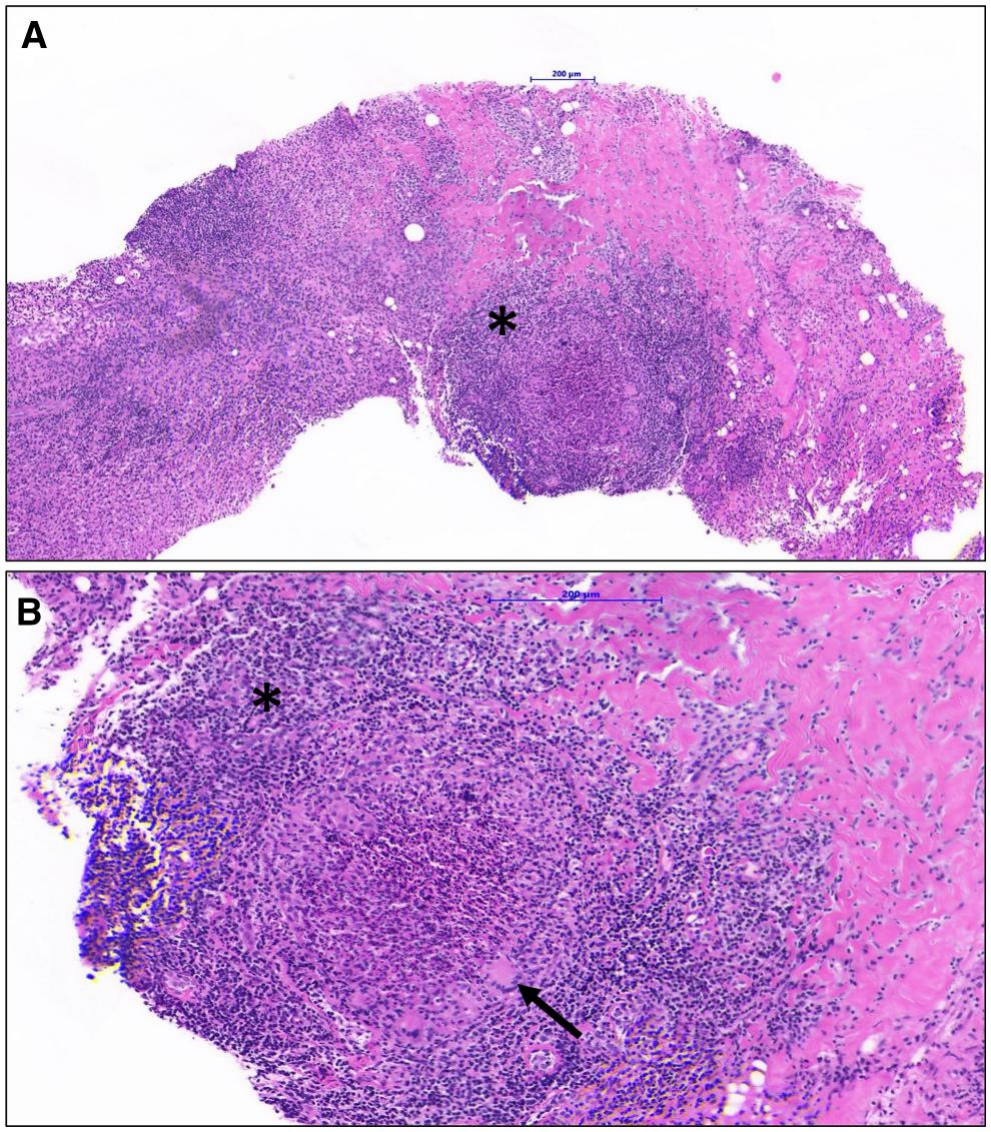
Abscessed granuloma without necrosis. (A) Diffuse infiltrate with marked lymphoplasmacytic inflammation around the granuloma and focal multinucleated giant cells with central abscessation (asterisk). (B) Focal multinucleated giant cells and occasional central microcysts (arrow). Diffuse infiltrate with marked lymphoplasmacytic inflammation and surrounding lobules (asterisk) (haematoxylin and eosin stain).

Histologic study of the specimen obtained with ultrasound-guided percutaneous core biopsy using a 14 G trucut needle revealed abundant granulomatous inflammatory areas with abscessed granulomas without necrosis, multinucleated giant cells, and lymphoplasmacytic inflammatory infiltrate (Figure 4). Immunohistochemistry and microbiology ruled out the presence of microorganisms (especially mycobacteria and Corynebacterium).

She underwent 3 months’ treatment with oral prednisone with progressively decreasing doses. Adherence to treatment was poor, and recovery was slow; after a good initial response, she had a few episodes of recurrence that resolved after rescue corticoid treatment. The patient is currently being followed up clinically with no drug therapy.

## Discussion

IGM is a rare inflammatory disease affecting women of childbearing age. To our knowledge, no reports of the development of IGM after COVID-19 vaccination have been published.

Although the pathogenesis of IGM is unknown, the most widely accepted hypothesis is that an autoimmune response in the lobules in the breast parenchyma secondary to an insult at the ductal level provokes a local inflammatory response in the connective tissue, with migration of macrophages and lymphocytes to the region giving rise to a noncaseating granulomatous response.^5^

The components of mRNA vaccines (lipid nanoparticles and antigenic proteins) stimulate the development of the immune response, reprogramming both the innate and adaptive responses, activating local and systemic proinflammatory processes and thus triggering related adverse effects,^6^ although the specific mechanisms involved are still under study.^7^

The clinical presentation of IGM is usually non-specific, consisting of signs and symptoms similar to those of infectious and inflammatory processes or even those of breast cancer, which can lead to diagnostic errors and delays in treatment. IGM is usually unilateral, but affects both breasts in some cases (1%-18%). The most common manifestation is a palpable mass.^1^ Pain of variable intensity is reported in 12%-44% of cases. Erythema is seen in one-third of cases.^1^ Another, less common sign is local oedema. Involvement of the nipple-areola complex is uncommon, although IGM can present with retraction or ulceration of the complex. In some cases, IGM manifests through sterile abscesses with or without fistulas to the skin. Nearly 30% of patients have ipsilateral axillary adenopathies.^1^

Ipsilateral axillary adenopathy is the most common adverse reaction to vaccination against COVID-19.^8^ A less common adverse effect is transient self-limiting oedema in the upper-outer quadrant of the breast/axillary tail of Spence ipsilateral to the inoculation site that resolves in 4-6 weeks.^9^ Our patient had an indurated palpable mass with mild erythema, mild pain, and local oedema; the nipple-areola complex was not involved.

From the clinical viewpoint, it is essential to differentiate IGM from periductal mastitis, a more common benign inflammatory process that presents as an indurated retroareolar mass, with hot reddened skin, oedema, breast pain, and sometimes fever. It is also crucial to differentiate IGM from inflammatory carcinoma, which usually occurs in older women, presenting with asymmetrical breast congestion, erythema, peau d’orange, and greater axillary involvement.^10^

Because they are widely available, mammography and ultrasound are the first-line imaging tests in cases where IGM or complications of vaccination are suspected; however, many of the associated findings are non-specific. Breast MRI enables a global evaluation of the breast, confirming the site, extent, and multicentricity of disease; however, in both dynamic sequences and diffusion-weighted sequences, the findings for IGM are non-specific and cannot differentiate it from malignant lesions.^11^

Given our patient’s young age, we first performed breast ultrasound. Beneath the palpable mass, ultrasound showed a heterogeneous area of breast parenchyma with areas of interstitial oedema and other ill-defined areas of posterior acoustic shadowing without a clear underlying mass. Ultrasound also revealed multiple suspicious lymph nodes with focal cortical thickening in level I of the ipsilateral axilla. These findings suggested a disease process other than transient oedema. Breast MRI done to better characterize the lesion showed regionally distributed non-mass-like pathological enhancement with progressive kinetics (type 3 curve) and intense restriction in diffusion-weighted sequences, associated with marked oedema in the adjacent parenchyma in T2-weighted sequences. Based on the clinical and imaging findings, core-needle biopsy specimens were obtained for histologic study.

IGM’s non-specific presentation requires a wide differential diagnosis that includes both benign conditions (infectious or idiopathic mastitis, iatrogenic lesions from surgery or radiotherapy, diabetic mastopathy, congestive heart failure, and prior trauma) and malignant disease (inflammatory carcinoma, primary breast cancer or metastatic lesions that obstruct lymphatic drainage, and lymphoma of the breast). In a recently vaccinated patient, it is important to rule out an adverse effect. In this context, transient breast oedema is the adverse effect that is most likely to require differentiation from IGM, especially in the absence of reactive lymphadenopathies. In our patient, the palpable mass with scant inflammatory signs and the ultrasound and MRI findings ruled out transient breast oedema.

The gold standard for the diagnosis of IGM is histologic study of core-biopsy specimens (fine-needle aspiration cytology is insufficient) showing an inflammatory lobular infiltrate with noncaseating granulomas.^1^ IGM is a diagnosis of exclusion after ruling out systemic autoimmune disease (vasculitis, sarcoidosis) and infections (tuberculosis, histoplasmosis, cryptococcosis, actinomycosis, and filariasis).^1^

After histologic confirmation of IGM, MRI is useful for providing information about the multiplicity, location, and size of the lesions; for identifying the formation of abscesses; for assessing the stability or progression of the lesions; and for monitoring the response to treatment.^12^

Several studies have suggested a potential correlation between COVID-19 vaccination and an increased risk of autoimmune diseases or autoimmune-related adverse reactions such as rheumatoid arthritis, systemic lupus erythematosus, Sjögren’s syndrome, autoimmune hepatitis, and immune-mediated nephropathy.^2^^,^^4^^,^^13^ In our patient, the time between the onset of IGM and vaccination against COVID-19 and absence of other causes that would explain the development of the disease suggests the possibility that COVID-19 vaccination could trigger it. This correlation is in line with hypotheses proposed by other authors to explain other autoimmune diseases appearing after vaccination against COVID-19. These authors point to the vaccines’ ability to catalyze an autoimmune response mediated primarily by T lymphocytes where molecular mimicry, the production of certain antibodies, and the activity of certain adjuvants would be the fundamental pillars supporting the development of autoimmune disease.^7^ It is clear that these proposed mechanisms are hypotheses and conjectures. Additional research is essential to confirm the role of these mechanisms in the development of COVID-19 vaccine-associated autoimmune diseases.

The treatment options for IGM range from watchful waiting to extensive surgical resection or mastectomy, with intermediate options including corticoids or methotrexate, antibiotics, and drainage of abscesses. Corticoids are the first-line treatment, and they result in complete recovery in up to 72% of cases.^1^ In our case, corticoid treatment resolved the initial episode, but poor adherence to treatment led to a torpid course of disease with some episodes of recurrence. The only consensus regarding imaging follow-up for IGM is periodic studies until the acute episode is resolved.^1^

## Conclusion

This case illustrates the development of IGM after vaccination against COVID-19. IGM could be considered in the differential diagnosis of breast lesions involving the axilla and breast in patients with a recent history of vaccination. MRI played an important role in the evaluation of the extent and possible complications. Given the non-specific clinical and imaging findings for IGM, biopsy is necessary to rule out infectious and malignant causes.

## Learning points

- Vaccination against COVID-19 has been associated with various autoimmune inflammatory processes.
- Like other autoimmune diseases, IGM might possibly be triggered by COVID-19 vaccination.
- The clinical and imaging findings of IGM can be similar to those of breast cancer.
- MRI is useful for the evaluation of the extent and possible complications of IGM.
- Given the non-specific clinical and imaging findings for IGM, biopsy is necessary to avoid diagnostic errors and delays in treatment.
